# Mitochondrial Pathophysiology on Chronic Kidney Disease

**DOI:** 10.3390/ijms23031776

**Published:** 2022-02-04

**Authors:** Patrícia C. Braga, Marco G. Alves, Anabela S. Rodrigues, Pedro F. Oliveira

**Affiliations:** 1 Department of Anatomy, Unit for Multidisciplinary Research in Biomedicine (UMIB), Institute of Biomedical Sciences Abel Salazar (ICBAS), University of Porto, 4050-523 Porto, Portugal; patriciacbraga.1096@gmail.com (P.C.B.); alvesmarc@gmail.com (M.G.A.); 2 Department of Nephrology, Santo António General Hospital (Hospital Center of Porto, EPE), 4099-001 Porto, Portugal; anabelarodrigues.nefrologia@chporto.min-saude.pt; 3 Nephrology, Dialysis and Transplantation, Unit for Multidisciplinary Research in Biomedicine (UMIB), Institute of Biomedical Sciences Abel Salazar (ICBAS), University of Porto, 4050-523 Porto, Portugal; 4 QOPNA & LAQV, Department of Chemistry, University of Aveiro, 3810-193 Aveiro, Portugal

**Keywords:** chronic kidney disease (CKD), electron transport phosphorylation (ETC) impairment, epithelial-mesenchymal transition (EMT), fibrosis, mitochondria, oxidative stress (OS)

## Abstract

In healthy kidneys, interstitial fibroblasts are responsible for the maintenance of renal architecture. Progressive interstitial fibrosis is thought to be a common pathway for chronic kidney diseases (CKD). Diabetes is one of the boosters of CKD. There is no effective treatment to improve kidney function in CKD patients. The kidney is a highly demanding organ, rich in redox reactions occurring in mitochondria, making it particularly vulnerable to oxidative stress (OS). A dysregulation in OS leads to an impairment of the Electron transport chain (ETC). Gene deficiencies in the ETC are closely related to the development of kidney disease, providing evidence that mitochondria integrity is a key player in the early detection of CKD. The development of novel CKD therapies is needed since current methods of treatment are ineffective. Antioxidant targeted therapies and metabolic approaches revealed promising results to delay the progression of some markers associated with kidney disease. Herein, we discuss the role and possible origin of fibroblasts and the possible potentiators of CKD. We will focus on the important features of mitochondria in renal cell function and discuss their role in kidney disease progression. We also discuss the potential of antioxidants and pharmacologic agents to delay kidney disease progression.

## 1. Introduction

Chronic Kidney disease (CKD) is a major global health concern that has high morbidity and mortality [1]. The continuous decline of renal function is closely associated with progressive aberrant accumulation of Extracellular matrix (ECM) proteins such as fibronectin and collagen that culminates in fibrosis. This progressive interstitial fibrosis is thought to be a common pathway for CKD [2]. Even though the extent of fibrosis does not correlate with the glomerular filtration rate (GFR) measure, it gives a hint to the future progression of the disease with greater certainty [3]. Mechanisms underlying fibrogenesis is a matter that has grown in scope and understanding in the present. However, the mechanisms in which this process can halt, or even reverse fibrosis remains poorly understood. 

Transforming growth factor-β (TGF-β) has been recognized as the canonical ligand and some strategies to halt fibrosis passes by controlling their precursors that drive pathological fibrotic responses [4]. Nevertheless, metabolic, and innate immune responses, both processes related to mitochondrial functioning, are emerging as plausible strategies to target CKD progression. Therefore, it is remarkably important to understand mitochondrial biology and pathophysiology for effective therapeutic options in renal diseases [5]. Reactive oxygen species (ROS) are not necessarily harmful to the cells. At moderated concentrations, ROS act as second messengers and regulate intracellular signal transduction pathways. A dysregulation between prooxidant/antioxidant equilibrium results in oxidative damage in cells, tissues, and organs [6,7], as the kidney a highly vulnerable organ, since it has a high energetic demand [8]. This review will discuss the role of Epithelial-Mesenchymal Transition (EMT) in interstitial fibrosis, a process that is associated with multiple kidney diseases that result in irreversible loss of renal function. The paradigm of EMT plasticity will be elucidated, as well as signaling mediators and potential therapeutic targets to avoid progressive kidney disease at earlier stages, even those that await deeper biochemical understanding. Furthermore, we will unveil the possible role of mitochondria dysfunction in the pathogenesis of kidney disease and summarize the recent development of mitochondria-targeted therapies.

## 2. Chronic Kidney Disease

The kidney has a high energy demand, due to its role in filtering the blood to remove accumulated toxins along with concentrating the urine to avoid dehydration [8]. To balance that activity, the kidney has an active repair capacity since they are able to restore in terms of function and structure after episodes of acute lesions. However, as it is a highly demanding organ, it is susceptible to multiple injuries that with time may result in major problems, such as culminating in a partial or even complete kidney failure. At these stages, the only solution is dialysis and ultimately, kidney transplant [9]. When it happens, kidneys fail to purify the body of metabolic end products, which are normally excreted into the urine and retained in the urea. Renal dysfunction can be determined when the glomerular filtration rate (GFR) is below 60 mL/min/1.73 m^2^ for more than three months and when albuminuria, defined as an albumin-to-creatinine ratio above 30 mg/g per day, is present [10]. Uremic retention is usually classified according to its removal pattern by dialysis and can be classified in three different groups, according to the reduced levels in patients at end-stage renal disease (ESRD): (1) small water-soluble compounds with a Molecular weight (MW) < 500 D, (2) middle- to large molecules, mostly peptides; MW > 500 D, and (3) protein-bound compounds with a low MW (<500), where these solutes are difficult to remove by any type of dialysis. In this last group, this type of retention is a trigger for kidney fibrosis in CKD [4]. 

Kidney disease can be divided into two major categories: acute kidney injury (AKI) and CKD. AKI is characterized by a fast renal function decline while CKD is linked to a gradual loss of renal function over time [11]. CKD is a worldwide health issue with consequences for the cardiovascular immune system which may lead to premature mortality [12,13]. Diabetes is the predominant pathophysiologic driver of CKD, although other conditions, such as hypertension, metabolic disorders, sepsis, ischemia/reperfusion injury, and exposure to nephrotoxins may also lead to CKD [14,15,16]. Depending on the initial site of injury, it will cause different subsequent pathologies. If there is a renal disorder that affects glomerular structure and function, it is characterized as glomerular fibrosis, whereas infections or proteinuria will lead to tubulointerstitial fibrosis [17]. Curiously, the development of fibrosis in the tubulointerstitium is the foremost predictor for the progression to ESRD in diabetic nephropathy (DN) [18]. DN is considered to be a syndrome [19] characterized by leakage of albumin, metabolites, and ions into the urine, alterations in the GFR, and a higher prevalence of stroke and cardiovascular diseases [20,21]. DN is one of the diseases that is the major cause of CKD and the absolute number of patients affected is still rising, demonstrating that this is a matter that deserves more attention, since diabetes unfortunately is also rising in astronomic proportions [22].

## 3. Fibrosis in the Kidney

In healthy kidneys, interstitial fibroblasts are responsible for the maintenance of renal architecture, which includes a balance between production and turnover of the ECM, mediating inflammatory processes [23,24]. However, prolonged injury in the kidney sponsors the progression of fibrosis in response to multiple nephropathies, including glomerulonephritis, spontaneous lupus nephritis, chronic allograft nephropathy, and DN [25,26,27]. The progression of fibrosis in the kidney affects 1 in 6 people globally annually per 100,000 individuals [28]. By definition, fibrosis is associated with significant alterations in the matrix, whereby functional tissue is replaced by connective tissue. In the fibrotic kidneys, the interstitial spaces are filled with predominantly type I and III collagen, and fibronectin [29,30]. Tubulointerstitial fibrosis is characterized by an excessive connective tissue accumulation during a reparative or reactive process that continues despite the resolution of the primary inflammatory process, leading to the gradual destruction of renal tubules and functional nephrons [31]. 

### Possible Origin of Fibroblasts

The source of interstitial fibroblasts remains a matter of debate. Some genetic mapping studies in mice pointed to a mesenchymal origin, resident in the kidney from nephrogenesis. These precursors are mostly attached to capillaries, specifically at branching points of the peritubular capillary plexus, and some investigators called these cells “pericytes” or “perivascular cells”, while others preferred to call these cells resident fibroblasts [31]. However, other studies identified that fibroblasts can be derived from leukocytes [32] or even that endothelial cells can be differentiated into “active” fibroblasts [33]. Theories that support the leukocytes as main precursors of fibroblasts rely on the fact that biomarkers used to detect fibroblasts, such as vimentin, labels macrophages and injured epithelial cells as well as fibroblasts. In support to this, Fibroblast-specific protein 1 (FSP-1), another common biomarker used to identify a supposed fibroblast formation, is also identified in macrophages in rodent models, but this expression is not observed in the fibroblasts [34]. Furthermore, the detection of collagen inside macrophages arrives in a possible ability of this type of leucocytes to produce elements for the pathological matrix. 

All of these data reveal that the real source of fibroblasts has to be recapitulated as well as the proper markers used to identify fibrogenesis processes. Additionally, the absence of epithelial to EMT as a source of fibroblast has been reformulated in multiple organs, and some conjectures were observed regarding leucocytes [35]. However, the contribution of circulating leucocytes to matrix-producing fibroblasts continues to generate discordance. Despite the advances that have been made, the studies were performed using an animal model, not in humans, and therefore the exact fibroblast origin population in human kidney disease remains unclear. Additionally, this gap of knowledge is mainly due to a lack of biomarkers available. Myofibroblasts possess the features of both fibroblasts and smooth cells and actively produce collagen in the repairing tissue [36,37]. Although the role of myofibroblasts is well documented, the basis of fibroblast origin, heterogeneity, and abundance in vivo is still a matter of debate with a lot of drawbacks. It is believed that fibroblasts are derived from multiple parental lineages, including endothelial, epithelial cells. A small number can also be derived from bone marrow cells [38]. In this review, we discuss the biomarkers available, including the mitochondria and metabolic fingerprints, and how they act to prevent kidney disease progression.

## 4. Epithelial–Mesenchymal Transition (EMT)

**Figure 1:**
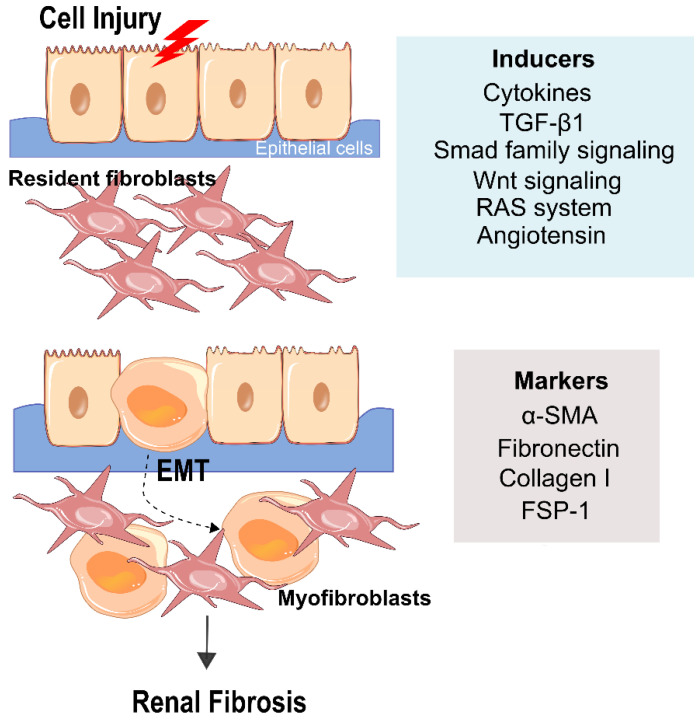
Epithelial and Mesenchymal transition. After injury, epithelial cells suffered a process called epithelial-mesenchymal transition (EMT), in which cells start expressing mesenchymal markers rather than epithelial markers. A wide number of factors have been pointed to as a potentiator of tubular EMT. The most potent inducer is Transforming growth factor-beta 1 (TGF-β1). Furthermore, signaling pathways that cooperate with TGF-β1 are also considered to be important mediators for EMT and consequently to renal fibrosis, such as Smad family, Renin-angiotensin system (RAS), and Wnt/β-catenin signaling. EMT is a highly coordinated process, characterized by the loss of cell-cell contact leading to cellular destabilization. Then, there is a de novo expression of mesenchymal proteins, such as α-SMA, and where it produced extracellular matrix proteins, including fibronectin, collagen I, and Fibroblast-specific protein 1 (FSP-1.) In the last phase of EMT, fibroblasts are activated and called myofibroblasts. These myofibroblasts share the same place that resident fibroblasts, whose proliferation is increased. This accumulation in the interstitium between tubules culminates in tubulointerstitial fibrosis.

In response to injury, epithelial cells undergo through EMT process, in which there is a presence of mesenchymal markers instead of epithelial markers [39]. EMT is a process highly coordinated composed of specific key events. The first phase of EMT is characterized by the loss of cell-cell contact and apical-basal polarity leading to a cellular destabilization. This destabilization is caused by the stimulation of tubular epithelial cells with cytokines such as epidermal growth factor (EGF), basic fibroblast growth factor (FGF-2) and TGF-β1, secreted mainly from infiltrating mononuclear cells, resulting in partial loss of epithelial membrane junction and tight junction proteins, such as E-cadherin and zonula occludin. The intermediated phase is related to the de novo expression of mesenchymal proteins, including α-smooth muscle actin (α-SMA) and synthesis of FSP-1 (Figure 1). At this point, there is an actin reorganization, with the reestablishment of the cytoskeletal proteins, and up-regulation of matrix metalloproteinases (MMP-2 and MMP-9) [27]. In the last phase of EMT transition, the fibroblasts are activated and become myofibroblasts, with an enlarged spindle-shaped morphology, with a higher capacity of cell migration and invasion into the tubulointerstitium [40].

Several cytokines and growth factors regulate EMT. Yet, it is believed that stimulation by cytokines is not enough to induce complete EMT. Several studies demonstrated that complete EMT only occurs when the integrity of the tubular basement membranes is disturbed [24,41]. Indeed, EMT is an extreme manifestation of epithelial cell plasticity but also a key event in the regenerative process of the kidney. EMT has emerged as a booster, yet highly debated, to renal fibrosis. Concerning epithelial cells, it is reasonable to hypothesize a switch to a mesenchymal phenotype since these cells during embryogenesis, has a mesenchymal origin. In 1995, Strutz and collaborators elucidated, for the first time, the EMT concept in adult solid organs, such as in the kidney [42]. Myofibroblasts, after injury, participated in the proliferation of resident interstitial fibroblasts. It is believed that cells such stem cells derived from bone marrow, epithelial cells, vascular smooth muscle cells, endothelial and pericytes, are associated as a source of myofibroblast development [32,43,44].

EMT participate in the pathogenesis of CKD progression in vivo and in certain conditions, it was reversible, i.e., mesenchymal cells acquired again epithelial phenotype. For that a dedifferentiation process is needed. Some shreds of evidence showed that renal tubular epithelial cells cope with damage and have the ability to acquire a stem-like phenotype, expressing CD24, CD133, and vimentin [45]. Like stem cells, these cells were able to proliferate and regenerate [46]. Another report suggests that an endogenous pool of stem cells that are present within the tubule mediates the repair and confer the epithelial phenotype back [47]. However, there is still a lot of controversy surrounding these theories. 

Renal tubular epithelial cells can differentiate, proliferate, and re-epithelize after being injured. Recent studies pointed to N-acetyltransferase-8 (NAT8) as a protective enzyme in the kidney. This enzyme is highly expressed at the kidney and is suggested to have a role in differentiation, and re-epithelization processes during kidney injury. Grácio and collaborators demonstrated that in prolonged injury, there was a decrease in NAT8 expression followed by a late rescue. This data indicated that NAT8 can be a possible target, once it maintains the regenerative potential when renal tubular epithelial cells dedifferentiate [48].

### Main Potentiators of EMT That Culminate in CKD

There is no doubt that EMT is present in the development and progression of the commonest renal diseases [49]. A wide number of factors have been suggested as crucial potentiators of tubular EMT. It was described that TGF-β1 has a critical role in the basic process of the EMT process, as it is considered to be the most potent inducer [50]. TGF-β1 is a profibrogenic cytokine that mediates the differentiation of fibroblasts into myofibroblasts. Mechanistically it suppresses collagen degradation and stimulates matrix-producing cell proliferation and collagen synthesis in the repairing tissue, leading to the fibrous formation [51]. TGF-β is secreted in an inactive state that requires processing before it can exert its effect, and meanwhile, it is stored on the surface of cells and the ECM, where it is transformed to active TGF-β [52]. At this point, TGF-β induces the differentiation of fibroblasts into myofibroblasts [53]. Along with this, TGF-β has pleiotropic effects, which simultaneously can regulate cellular proliferation, migration and differentiation, modulation of the immune system [54], and stimulation of oxidative stress [55]. In mammals, there are three TGF-β isoforms, nonetheless, the isoform TGF-β1 is the most associated with renal fibrosis [56]. Indeed, the kidney is highly susceptible to its overexpression [57]. It is widely expressed in all kidney cells, particularly in glomeruli [51], and even expressed in macrophages that invade the kidney [52].

In addition to the molecular pathways between the cooperation of TGF-β1 and cytokines that drive EMT, the Smad family of transcriptional activators are considered to be the most important mediators of TGF-β1 signaling. The Smad family is comprised of three different subgroups considering their role in TGF-β family signal transduction: Receptor-regulated Smads, known as R-Smads (which includes Smad 1, 2, 3, 5 and 8), common Smad4 (Co-Smad), and Smads 6 and 7, considered to be Inhibitory Smads (I-Smad). The role of Smads is intimately related to the progression of CKD. The most important are Smads 2 and 3, which control TGF-β in renal fibrosis, being fibroblasts highly activated in DN, hypertensive nephropathy, and obstructive kidney disease [58,59,60]. Smad 7 controls renal inflammation [61]. TGF-β signaling is initiated with serine/threonine kinase receptor oligomerization and R-Smad phosphorylation. After that an R-Smad/Co-Smad complex is translocated into the nucleus to control transcription of TGF-β responsive genes. I-Smads are responsible for dissipating TGF-β signaling by blocking R-Smads [62,63,64]. Multiple in vivo studies of different renal-related diseases revealed that after silencing Smad 3, the fibrosis process was alleviated, suggesting that Smad 3 can be a critical pathogenic mediator of TGF-β/Smad signaling in renal fibrosis [65,66,67,68]. In concordance with these studies, Li and collaborators demonstrated that MAPK-Smad signaling can directly activate Smads 2 and 3 to mediate diabetic injury [58]. Moreover, Smad 3 is associated with fibrotic genes, such as collagen [69,70]. Moreover, Bone morphogenic protein (BMP-7), a member of the TGF-β superfamily, can also inhibit TGF-β induced EMT in Smad dependent fashion [71] and Smad-independent pathways such as ERK and MAPK through Smad 1 and 5 activation [72,73,74]. It is highly expressed in the kidney, and in experimental animal models revealed to be capable of halting and reverting renal fibrosis [75]. Additionally, BMP-7, through Smad signaling, plays a protective role in podocyte differentiation [76]. 

Tubulointerstitial fibrosis, as previously mentioned, is associated with hypertension, inflammation, and activation of some signaling pathways and systems, such as TGF-β1/Smad and Wnt/β-catenin and renin-angiotensin system (RAS) [77,78]. They are closely linked as they form a vicious cycle in which the RAS stimulate tubulointerstitial fibrosis by triggering TGF-β/Smad and Wnt/β-catenin signaling. In a limiting renal blood flow situation, tubulointerstitial fibrosis augments arterial blood pressure and consequently activates RAS, which in turns activates TGF-β/Smad and Wnt/β-catenin signaling [79]. Wnt/β-catenin is activated in multiple kidney diseases, such as DN, polycystic kidney disease and obstructive nephropathy [80,81]. Furthermore, β-catenin has a critical role in the kidney in terms of controlling the expression of the intrarenal RAS. A RAS cascade event starts with the conversion of angiotensinogen into angiotensin (Ang) I through renin. Ang I is subsequently converted to Ang II, the major effect of the RAS, through the angiotensin-converting enzyme (ACE). Renin is the rate-limiting step in RAS activation [82]. All the components of RAS are expressed in kidney tissues, and its activation is intimately related to kidney injury [77,83]. Ang can cause pro-fibrotic event in proximal tubular epithelial cells, podocytes, and mesangial cells of the kidney [79]. Multiple studies already exposed the interaction between Ang II and TGFβ-1 that influences ECM production [79]. It also induces EMT in tubular epithelial cells [84], by stimulating the collagen and fibronectin expression [85]. Therefore, simultaneous blockade of RAS activation and inhibition of TGF-β/Smad and Wnt/β-catenin signaling present are cornerstone therapies against the progression of CKD. 

FGF-2 is a mitogen for renal fibrosis. It induces proliferation in primary cortical fibroblasts and stimulates the expression of fibroblastic markers [39]. Additionally, the induction of EMT is magnified when conjugated with TGF-β1 [86]. Connective Tissue Growth Factor (CTGF) is a modulator of TGF-β and MBP-7 antifibrotic signals. CTGF binds to TGF-β1 and potentiates myofibroblast activation, fibronectin accumulation, and α-SMA expression [87]. It was initially reported in renal cells in 1997, and over the years has been correlated with the degree of tubulointerstitial fibrosis [71,88]. In multiple human and animal models of kidney fibrosis, including DN, CTFG has been found overexpressed [89,90]. The anti-CTGF antibody, FG-3019, was described to be effective and well-tolerated in phase I clinical trial for the treatment of microalbuminuria DN [91].

## 5. The Possible Mechanisms to Delay Fibrosis Progression—Pharmacological and Metabolic Approaches

In healthy kidneys, there are endogenous signals that maintain the normal epithelial phenotype in tubular cells and keep homeostasis. However, with the progression of CKD, these homeostatic mechanisms are disrupted, resulting in fibrosis progression. Given the impact of EMT in CKD progression, there is an urgency to find novel ways to delay, inhibit or even reverse this process. To cope with this problem, some strategies have been developed to slow down or reverse the fibrogenesis process.

### 5.1. Mechanisms Associated with Signaling Pathways

A pharmacologic approach to delay the progression of fibrosis through TGF-β1 blockage has not revealed the best therapeutic approach. Indeed, LY2382770, an anti-TGF-β1 antibody that blocks all the three forms, in phase II study revealed no efficacy [35]. Fresolimumab is a human monoclonal antibody able to neutralize the isoforms of TGF-β. It was administrated in a single infusion in patients with primary focal segmental glomerulosclerosis. It has advanced to phase I clinical trial; however, its effectiveness was not demonstrated [92]. In phase II clinical trial, another human monoclonal antibody against TGF-β1, is performed in diabetic patients. Nonetheless, no inhibitory effects were observed [93].

Under the importance of Smad signaling in kidney disease, it is valuable to target these downstream mediators of TGF-β signaling. Selective Smad 3 inhibitors have demonstrated the ability to block excessive ECM production and EMT, halting the development of diabetic renal fibrosis. Indeed, an in vivo study with streptozotocin (STZ)-induced diabetic nephropathy mice, demonstrated to be able to halt the development of diabetic renal fibrosis [94]. In this study, the authors found that a specific inhibitor of Smad 3 was able to inhibit TGF-β1. However, no significant changes were observed on albuminuria levels. This study demonstrates, again, the importance of understanding the molecular mechanisms that are behind slight processes that can advance in kidney disease, more specifically in DN, once albuminuria is no longer a biomarker plausible to detect this pathology, specifically when diabetes disease is present. 

Wang and his collaborators used Human Kidney (HK-2) cells and mice with unilateral ureteral obstruction (UUO) and tested novel tetracyclic triterpenoid compounds (PZC, PZD, and PZE), in which they act on the inhibition of the RAS, TGF-β/Smad, and Wnt/β-catenin signaling. These compounds were revealed to selectively inhibit TGF-β1 and Ang II-induced Smad3 phosphorylation by blocking the interaction of TGF-β1 with Smad3 [95]. Moreover, ACE2, was discovered as a homolog of ACE, with a higher catalytic efficiency for degrading Ang II than ACE [96]. This enzyme is highly distributed in the kidney, especially in the brush border of proximal tubules [97]. However, the distribution is altered when kidney disease is present. Notably in DN, the RAS system is a contributor to the progression of the disease. ACE2 expression, has been shown to be altered in diabetes. Its gene deletion expands fibrosis, albuminuria, and glomerular hypertrophy in Akita mice [98]. Additionally, the evaluation of Ace2 in urine could provide a better picture of renal injury and disease progression. It was noticed that ACE2 in urine was higher in patients with CKD compared to healthy patients, and even higher in patients with DN, compared to patients with other renal diseases [99]. Furthermore, patients with urinary ACE2 have been described as variable and can be dependent of the stage of the disease, once it is more present in patients with longstanding diabetes type 1 and CKD that with patients with only diabetes [100]. Collectively, these studies demonstrated that ACE has a renoprotective role in experimental models of diabetes and can act as a promising early clinical marker for DN. The administration of human recombinant ACE has been demonstrated to prevent Ang II-mediated hypertension and renal injury [101,102] and reduce the progression of DN in mice [103]. Clinical trials are already ongoing, demonstrating positive effects, where the administration was well-tolerated in healthy individuals [104] and multiple pharmacologic solutions were tested in order to modulate the RAS system and decrease the progress of renal diseases [105]. 

Another possible approach found was through the inhibition of signaling pathways that are intimately related to immune signaling that activate the pathological fibroblast. Genetic studies already demonstrated that there is a predisposition to fibrotic kidney disease through dysregulation of the innate immune system. Some polymorphisms in Toll-like receptor 4 (TLR4) [106], Interleukin (IL) IL1β, and IL1 [107,108] are associated with CKD. This correlation unveils the possibility to dampen pro-fibrotic responses and inflammation, without blocking downstream signaling pathways that have homeostatic functions such as NOTCH, WNT, TGFβ, or HEDGEHOG. One example was the inhibition Janus kinase-signal transducer and activator of transcription pathway (JAK-STAT) in DN, through Baricitinib. This study demonstrated a reduction in proteinuria levels [35]. Receptor tyrosine kinases (RTKs) are important players in the pathogenic process of kidney fibrosis. In 2019, Park and his collaborators revealed that the absence of macrophage-stimulated 1 receptor (RON), a RTK receptor that attenuated the expression of EMT and fibrosis-related proteins through control of Src and Smad pathways in HK-2 and fibroblast (NRK49F) cells [109]. Among other studies, RTK inhibition aid in the prevention and treatment of kidney fibrosis. Imatinib, which is a platelet-derivated growth factor receptor (PDFGR) inhibitor, diminished pathological alterations in different models of CKD [110]. Furthermore, AG1296, the inhibitor of fibroblast growth factor receptor (FGFR) can block the proliferation of kidney fibroblasts [39]. RTK is also linked to TGF-β signaling. That is shown when epidermal growth factor receptor (EGFR), another RTK related to kidney fibrosis, is inhibited, and lower fibrogenesis mediated by TGFβ occurs [111]. 

Angiotensin II, as previously described, can induce TGF-β1 synthesis in tubular epithelial cells and fibroblasts. Some studies claim that blocking RAS can delay the progression of renal disease. Losartan [112] and Captopril [113] were already described as a possible pharmaceutical solution to delay renal diseases such as chronic allograft nephropathy and diabetes mellitus. BMP-7 is described to have the ability to reverse EMT, through reinduction of the epithelial cell adhesion molecule, E-cadherin. Intraperitoneal injection of human recombinant BMP-7 in mice with nephrotoxic serum nephritis, halt the disease progression [71]. There are currently no antifibrotic drugs approved for the treatment of CKD, representing a major unmet medical need [114]. 

In recent years, metabolic alterations are recognized as an important pathogenic process that underlies fibrosis. Therefore, metabolically targeting therapies could become important strategies for fibrosis reduction [115].

### 5.2. Metabolic Targeting Therapies

One possible approach is to follow the disordered metabolism of carbohydrates, lipids, proteins, and hormones. Indeed, ECM is intimately related to glucose metabolism. Targeting glucose metabolism has demonstrated interesting results in halting fibrosis. Ding and collaborators developed a study where they administered 2-Deoxy-d-Glucose (2-DG), a hexokinase inhibitor in rat renal fibroblast cells, which resulted in a diminished expression of TGFβ-associated biomarkers [116]. Furthermore, several clinical outcomes demonstrate that glucagon-like 1 receptor agonist (GLP-1 Ras) have beneficial effects on renal outcomes, being particularly beneficial in terms of oxidative stress and inflammation. Mechanistically, GLP1-Ras improved glucose metabolism by increasing insulin secretion and suppression of glucagon [117]. Within this class of pharmacologic options, some clinical trials showed promising results in diminishing albumin. For instance, the LEADER trial, which involves liraglutide, demonstrated that 52 weeks of treatment led to an increase in glomerular filtration rate and reduction of albuminuria [118]. The SUSTAIN-6 trial was a double-blind trial in which each type two diabetes patient received 0.5 mg once-weekly of semaglutide, and this drove a reduction in new onset macroalbuminuria when compared to the patients that received the placebo [119].

DN, an in vitro study, using HK-2 cells, demonstrated a new mechanism for the amelioration of renal fibrosis. Here, the authors used exendin-4, and it was able to down-regulate miR-192 expression during high glucose treatment. Thus, GLP1R seems to be a key in the regulation against renal fibrosis during diabetic kidneys [120], resulting in a decrease in mesangial and endothelial dysfunction, as well as a decrease in podocyte loss [121]. 

Furthermore, the role of the kidneys in glucose homeostasis gained attention once glucose transport through sodium glucose transporter-2 (SGLT2), consumed a high proportion of oxygen and ATP in the kidney [122]. In patients with long-standing diabetes, inhibition of SGLT2 by empagliflozin administration halted the advance of renal disease [123]. Along with this, inhibition of SGLT2, in rats with early diabetes, decreased tubular Na+/glucose cotransport, which increased the sodium delivery to the macula densa, resulting in a diminished glomerular hyperfiltration [124].

Targeting fatty acid metabolism and/or receptors also yielded beneficial effects in reducing fibrosis. Free fatty acid receptor 1 (FFA1R) and GPR84 are G-protein-coupled receptors that modulate metabolism through insulin secretion and cellular respiration. In Ffa1r−/− or Gpr84−/− knockout mice used as models of kidney fibrosis, FFA1R revealed to have a protective effect against renal fibrosis, whereas GPR84 had the opposite effect [125]. After this discovery, in this study, the authors used PBI-4050, a synthetic fatty acid ligand that has agonist affinity to FFA1R and antagonist affinity to GPR84. Therefore, the authors concluded that this compound significantly attenuated fibrosis, being effective for managing inflammatory and fibrosis-related diseases observed in multiple fibrosis models [125]. Additionally, managed carnitine *O*-palmitoyltransferase 1 (CPT1) may be useful for preventing and treating CKD. Human and mouse models with tubulointerstitial fibrosis had lower expression of key enzymes and regulators of fatty acid oxidation (FAO) and hence higher lipid deposition. Kang and collaborators used a synthetic compound, C75, which is a CPT1 activator and fatty acid synthase inhibitor and demonstrated that it was able to preserve renal cell viability and decreased renal fibrosis in human kidney tubule samples [126]. 

Endothelin-1 (ET-1) is a vasoconstrictor peptide with a role in hypertension and kidney injury [127,128]. It promotes proteinuria and has been implicated in renal inflammatory and fibrotic pathways [129]. In addition, ET-1 is increased in diabetes, being an increased risk of renal disease [130]. In diabetic rats, administration of atrasentan or avosentan, an ETA receptor blockage, reduced albuminuria and renal fibrosis [131,132]. Unfortunately, the administration of avosentan in combination with RAS blockade in patients with DN, after 4 months of treatment has not successfully progressed due to excess cardiovascular adverse events [133].

### 5.3. Natural Approaches

Natural compounds were already tested in CKD, however, all of them revealed an insufficient quality. Emodin is a natural extract from rhubarb and has beneficial effects on renal function in vivo [134]. To improve the low water solubility and kidney nonspecificity, Huang and collaborators encapsulated emodin in a self-micro emulsifying drug delivery system, which resulted in an improvement of its oral bioavailability, leading to an inhibition of fibronectin, TGF-β1, and intercellular adhesion molecule 1 expression [135]. In sum, drug delivery and therapeutic effects need to be recapitulated, once some pharmacological approaches need the administration of high doses daily, which leads to drug-related toxicity side effects. 

Celastrol is a triterpene derived from traditional Chinese medicine, with potent anti-fibrotic proprieties. It is known to suppress cardiac [136] and pulmonary fibrosis [137]. Tang and his collaborators explored its possible effects on renal fibrosis development. They concluded that celastrol was able to attenuate the progression of renal fibrosis by up-regulating cannabinoid receptor 2 expression, which is an anti-fibrotic factor, through Smad3 signaling inhibition on HK-2 cells [138].

Low Vitamin D level is correlated with patients with CKD. It is linked to RAS activation and podocyte injury [139,140]. Additionally, it is believed to have a key role in EMT transition [141]. Vitamin D may also have positive effects on oxidative stress, through restoration of nuclear factor erythroid 2-related factor 2 (Nrf2) levels [142]. Additionally, in the VITAL study, type 2 diabetics randomized to a synthetic D2 agonist (paricalcitol) were able to reduce albuminuria, in a 24-week treatment [143].

### 5.4. miRNA Approaches and Others

Hormonal therapy has been recognized to have a beneficial role in fibrosis. As happens in lung fibrosis [144] and skin fibrosis [145], administration of thyroid hormone through aerosolized active thyroid hormone (T3), prevented renal fibrosis. In this model, T3 increased the expression of the microRNA miR34a, inhibiting the EMT induced by TGBβ-1 [146]. This study, along with others, demonstrated the possibility of improving the delivery of miRNA or siRNA into the kidney. The common modality for the administration of siRNA is through intravenous injection [147]. In mice, intravenous administration of Smad4 siRNA prevented folic acid-induced in renal fibrosis process by inhibiting α-SMA expression [147].

Gene therapy using Smad microRNA is another solution. miR-21 [148], miR-29 [149], and miR-192 [150] expression are Smad3-dependent and acted to decrease the fibrotic marker levels. Indeed, inhibition of miR-21 in unilateral ureteral obstruction (UUO) kidneys decreased collagen deposition and reduced fibronectin, α-SMA, and plasminogen activator inhibitor 1 and increased the levels of TGF-β in injured kidneys [151].

One curious way to take advantage of miRNA-based therapeutics is when is transported via exosomes. miR-let7c is down-regulated in fibrogenesis in vivo and in vitro [152]. Wang and collaborators injected mesenchymal stem cells overexpressing miR-let7c to the renal parenchyma via exosomes and resulted in an attenuation of kidney injury, by down-regulating the expression of collagen, MMP9, and TGF-β1 in NRK52E cells and UUO kidneys [153]. 

Another possible approach is using Lysozyme (LZM) for kidney-specific delivery for targeting kidney cells [154]. This approach takes advantage of the fact of being a low molecular weight protein, which passes freely through the glomerulus during filtration and subsequently reabsorbed by proximal tubular epithelial cells. Allied to this, it is biodegradable and considered non-immunogenic [155]. However, this technique needs improving since it has cardiovascular side effects [156].

Moreover, stem cells revealed potential benefits in DN. Park and collaborators developed a study, in which they treated diabetic Sprague-Dawel rats with human umbilical cord blood-derived stem cells (hUCB-SC) had treatment for four weeks. Intravenously administration of hUCB-SC reduced proteinuria, renal fibronectin, α-SMA and E-cadherin down-regulation [157]. Notwithstanding, some studies point out that stem cells may be a mechanism to reduce kidney disease progression, but there is no evidence on this approach in human studies yet [158].

Human kidney organoids that derive from stem cells are pointed out as promising tools for the personalized evaluation of factors important for the development and prevention of fibrosis. However, these organoids fail in some aspects, such as a lack of blood supply, in glomerular filtration, they do not have immune responses, and hence, they may recapitulate only some of the molecular and cellular responses seen in adult kidneys after AKI [159]. Although several studies were successful at the pre-clinical level, only limited advances have been made so far in the translation of these findings to the level of patient treatment [89].

Taken all the strategies adopted above together, there are still mechanisms to unveil.

Furthermore, there are some tubular biomarkers in urine used to evaluate the development of CKD, such as KIM-I [160,161], NGAL [162], l-FABP [163], and Cystatin C [164]. However, these biomarkers have been inconsistent, with no valuable results on the prediction of CKD in humans. It should be noted that several clinical trials are already underway, to circumvent kidney diseases, such as DN, as well as all the mechanisms associated with it (i.e., fibrosis). However, they all have a flaw. The therapies used, as detailed in Table 1, are adapted from other pathologies, have a slight effect or take a long time before that effect is pronounced [158]. Thus, the search for more reliable renal biomarkers, with more sensitivity and specificity is extremely important for early prediction of the onset and monitoring of the progression of kidney-related diseases. 

In the past few years, more attention has been pained to the role that mitochondria can have on CKD, and how its dysregulation can be related to a start point of the disease.

## 6. Energy Generation in the Kidney

**Figure 2:**
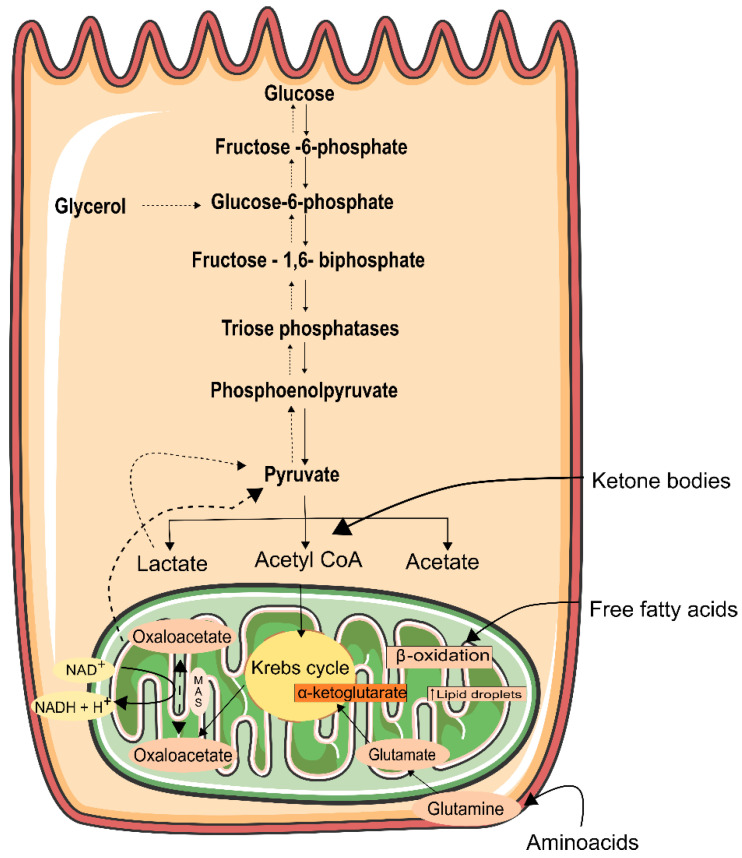
Schematic overview of the major metabolic pathways in renal epithelial cells. Principal substrates used to produce ATP. In renal cells, the uptake of free fatty acids, ketone bodies, glucose, and glutamine lead to increased oxygen consumption and ATP production, through ATP synthase. Glucose, in distal tubules in the healthy kidney, is metabolized to glucose-6-phosphate which is converted into Fructose-1,6-biphosphate. Then, Fructose-1,6-biphosphate is cleaved into two phosphorylated three-carbon compounds glyceraldehyde 3-phosphate and dihydroxyacetone phosphate), known as triose phosphatases. Afterward, glyceraldehyde 3-phosphate is converted into phosphoenolpyruvate and finally into pyruvate. Free fatty acid oxidation is a more efficient substrate to use than glucose in terms of energy production; however, in diabetic nephropathy disease, some complications occurred, i.e., increasing of lipid droplets. Within the cortex, ketones bodies are also a substrate used for ATP production. Glycerol becomes an important gluconeogenic precursor in diabetes. It is broken into glucose-6-phosphate and then be transformed into pyruvate and continues to follow the same path as glucose metabolization. Gluconeogenesis (up dashed arrows), which occurs in proximal tubules cells, needs ATP to produce glucose, which can be synthesized from lactate, and glycerol. Glutamine is degraded and converted to glutamate, which can be transaminated via glutamate-oxaloacetate-transaminase or glutamate-alanine-transaminase, which bot reaction yield α-ketoglutarate, an intermediate of Krebs cycle. Pyruvate resulted from lactate, enters into the mitochondria, and is converted to oxaloacetate. Oxaloacetate, in the cytoplasm of the mitochondria, can be reduced to malate and be exported out and/ or in the cytoplasm. Here, through malate aspartate shuttle (MAS), first, malate is oxidized to oxaloacetate and then converted to phosphoenolpyruvate and subsequently converted to fructose-6-phosphate. After a phosphatase, fructose-6-phosphate is dephosphorylated, which results in glucose-6-phosphate, which then is converted into the release of glucose.

As stated previously, the kidneys have a high density of mitochondria and thus a high consumption of oxygen. This oxygen is metabolized within the mitochondria by oxidative phosphorylation (OXPHOS), through the electron transport chain (ETC), to produce ATP [165]. In a very simple way, a wide range of fuels (glucose, fatty acids, and amino acids) are converted into molecules of ATP. In this process, oxygen acts as an electron acceptor in highly regulated reactions carried out by protein complexes I–IV. The ATP synthase (also called complex V) couples the electrochemical proton gradient generated by electron transport to ATP synthesis. The protons that are pumped into the intermembrane space as electrons move through complexes I, II, and IV to ATP synthase (complex V) to drive the conversion of ADP to ATP [165] (Figure 2). Additionally, the Krebs cycle (TCA) cycle plays pivotal roles in mitochondrial energy production [166]. TCA is responsible for the production of the following coenzymes in the mitochondrial matrix, which englobes three molecules of nicotine adenine dinucleotide (NADH), and one molecule of flavin adenine dinucleotide (FADH_2_), which is converted from pyruvate after the glycolysis pathway [167].

The fuel preferences are different and tend to mirror ATP demands at these sites. In the healthy kidney, especially in the thick loop of Henle, glomerular cells, and in the vasculature, about 90% of renal ATP production occurs via OXPHOS with yielding of 36 molecules of ATP per glucose molecule. Furthermore, ATP generated via OXPHOS, is used for the reabsorption of glucose, ions, and other metabolites from filtered urine [168]. OXPHOS machinery can also be used for hormone and protein synthesis and glomerular filtration, including contraction of mesangial cells and podocytes [169]. However, in a more hypoxic renal medulla, what prevails is anaerobic metabolism, where the ATP profit is only two molecules per glucose [170]. Free fatty acid (FFA) oxidation is another fuel that can be used in kidney cells, particularly in the tubulointerstitial compartment (excluding the glomeruli). Oxidation of palmitic acid produces 106 molecules of ATP per molecule of substrate, being more efficient than glycolysis [170]. The accumulation of FFA in AKI and DN exhibit abnormalities in lipid metabolism, increasing the formation of lipid droplets inside the cells [171]. Podocytes can be damaged due to FFA accumulation, which can sense disruption of the glomerular filtration barrier, through the free passage of FFA [172,173]. In rodents, high circulation of FFA revealed susceptibility to renal injury [174]. Lipid-lowering therapies are considered to have protective effects concerning kidney fibrosis [126]. Nevertheless, these mechanisms are controversial once they can be considered to be pathogenic or compensatory responses to renal injury [175,176,177]. As happens with FFA, ketone bodies require higher amounts of oxygen to produce ATP [178]. Notwithstanding, ketone bodies are an important fuel for OXPHOS within the kidney cortex [179]. However, little is known about their contribution to the kidney. The use of ketone bodies along the nephron is reflected in the mitochondrial density, which is high at this site [180]. Moreover, increased ketogenesis also occurs in glomerular cells in diabetes; however, the rate of intake of ketones in the kidney is not altered in diabetes [181], and so ketone bodies may be an endogenous process, with no relationship with diabetes, but with renal injury only. However, more studies are needed, as well as the contribution of these metabolites and what is the consequence of its increase in mitochondrial dysfunction in the kidney.

Proximal tubular cells need ATP to produce glucose via gluconeogenesis [182] (Figure 2), in which glucose is synthesized from precursors such as lactate, glutamate, and glycerol [183]. Glutamine is the most common amino acid in the human body, and in the kidney, makes a higher contribution to gluconeogenesis than does alanine [184]. 

Renal gluconeogenesis, in normal individuals, represents nearly 60% of endogenous glucose release during the post-prandial period, while this proportion is half reduced in a patient with type 2 diabetes [183,185].

In diabetic kidneys, these values are exacerbated via stimulation of the malate aspartate mitochondrial shuttle [186], increasing the generation of NADH, FADH2, and ATP, and ultimately increases in renal gluconeogenesis [187]. Moreover, glutaminase, the enzyme responsible to convert glutamine into glutamate [178], is expressed at high concentrations in the tubular compartment of the kidney [188]. Nonetheless, in the diabetic kidney, the impairment of autophagy increases the use of less favorable fuel sources. For instance, glucose is used by proximal tubular cells and fatty acids are used by glomerular epithelial cells, leading to glucotoxicity and lipotoxicity, respectively [189].

## 7. Mitochondrial Function in the Kidney

Kidney disease is a worldwide health issue with an unpredictable prognosis that lacks therapeutic options. Mitochondrial biogenesis, fission, fusion, and mitophagy form an essential axis of mitochondrial quality control, to the optimal production of ATP. Mitochondria is responsible for the regulation of cellular oxidative stress, apoptosis-signaling pathway, aging, in all cells and tissues, and calcium homeostasis [168,190]. Renal diseases, such as AKI and DN, can induce an imbalance in mitochondrial homeostasis [168]. 

Renal tubular cells, which constitute nearly 90% of the outer kidney cortex, have important functions associated, such as reabsorption of nutrients, secretion of the waste from the blood, regulation of the fluid, and electrolyte balance [191]. These functions require a high expenditure of energy, in which mitochondria plays a vital role in energy production. Mitochondria must maintain their normal function to cellular homeostasis in normal organs, and the kidney is not an exception [192,193]. Indeed, the kidney is considered, among other organs, to be one of the most energy-demanding in the human body. In fact, it has the second-highest mitochondrial content and oxygen consumption [194,195]. Within the kidney, the renal proximal tubule accounts for the greatest proportion of oxygen consumption, being a highly ATP-dependent process [196]. The requirement of ATP in glomerular cells is lower since it required lower energy for filtration and other passive processes [197]. Persistent tubulointerstitial injury, with time, is directly associated with mitochondrial dysfunction [198,199]. To further support this, a report in the early stages of DN, in a rat model, described ultrastructural changes in renal proximal tubular mitochondria, which were correlated with disturbance in the main function of renal tubular cells [200].

## 8. Oxidative Stress and Electron Transport Phosphorylation Impairment in Kidney Function

### 8.1. Oxidative Stress (OS) Percussions in the Kidney

An imbalance between reactive oxygen (ROS) and nitrogen (RNS) species and cellular antioxidant defenses is termed oxidative stress (OS) [201]. OS plays a critical role in CKD progression, by inducing glomerular and tubular damage or is indirectly associated with inflammation, hypertension, and/or endothelial dysfunction [202]. ROS disturb regulatory mechanisms of the kidney, such as RAS system, tubular glomerular feedback, and myogenic reflex in the arteriole. Therefore, the kidney fails to compensate for water-electrolyte and acid-base imbalances, resulting in an additional increase of OS [203].

Sources of ROS formation include the mitochondrial electron transport chain, catecholamine oxidation, metabolism of arachidonic acid, endothelial cells (xanthine oxidase reaction), and inflammatory cells (myeloperoxidase and nicotinamide adenine dinucleotide phosphate (NADPH) oxidase) [202]. NADPH oxidase 4 (NOX4) is the most important isoform, located in renal fibroblasts, renal tubules, glomerular mesangial cells, and podocytes in the kidney and in the membrane of mainly endothelial cells and fibroblast in the vasculature [201]. The enhanced Nox4 expression leads to an increase in ROS levels. These ROS reduced cytochrome C oxidase activity and caused mitochondrial dysfunction, by inhibiting mitochondrial oxidative phosphorylation [204]. Several kidney cells are under changes when OS imbalance occurs. For instance, high glucose induces intracellular ROS in proximal tubular epithelial cells. In addition, ROS are induced in glomerular mesangial by advanced glycation end products and cytokines [205]. High levels of glucose induce micro-vesicle generations through ROS/Nox4 pathway in mouse podocyte clones cells. The treatment with *N*-acetylcysteine (NAC), a powerful antioxidant, diminished podocyte injury by increasing nephrin expression and inhibiting apoptosis [206].

Indeed, ROS production in response to several stimulus, activates the NADH/NADPH oxidase system in renal cells. Nicotinamide adenine dinucleotide (NAD+) is essential not only for the harvesting of energy in the form of ATP from different fuels, but also to protect the kidney tubule against acute stressor. NAD^+^ augmentation may alleviate AKI triggered by ischemia-reperfusion (IRI), systemic inflammation, and toxic injury. Indeed, data from experimental models and clinical studies have pointed to NAD+ homeostasis as a key factor of kidney health [167]. Metabolic analysis of mouse urine samples identified an increase in quinolinic acid following IRI. Quinolinic acid serves as a precursor for NAD^+^ biosynthesis. Accumulation of this acid in the urine suggested a reduction in the levels of the enzyme that uses QPRT substrate. This reduction led Mehr and collaborators to suggest that reduced QPRT expression increased AKI susceptibility in mice [207]. Moreover, in another study, quinolinic acid and tryptophan were measured in urine samples obtained from 215 patients within the first 24 h of intensive care unit admission. Fifty-one patients developed AKI and the increased urinary quinolinic acid: the tryptophan ratio was independently associated with increased risk of AKI, which raises the need for renal replacement therapy and adverse outcomes [167]. The NAD^+^ metabolism suggests having a protective effect on exogenous Nam, a member of the water-soluble vitamin B family, in a murine UUO model of renal fibrosis. Nam treatment reduced multiple outcomes that follow UUO, including fibrosis, apoptosis, and tubular atrophy. It inhibited the expression of TGF-β1. This result may target exogenous Nam administration as a potential therapeutical approach to halt CKD progression [208].

Morphological abnormalities and loss of function in mitochondria, through swelling and fragmentation, results in ROS production such as superoxide (O_2_^−^) and hydrogen peroxide (H_2_O_2_), leading to OS and inflammation, mechanisms that trigger the progression of renal fibrosis [209,210]. Moreover, OS is predominantly induced by excessive cytoplasmic and mitochondrial ROS emission [190].

### 8.2. Mitochondrial Oxidative Phosphorylation Impairment in the Kidney

**Figure 3:**
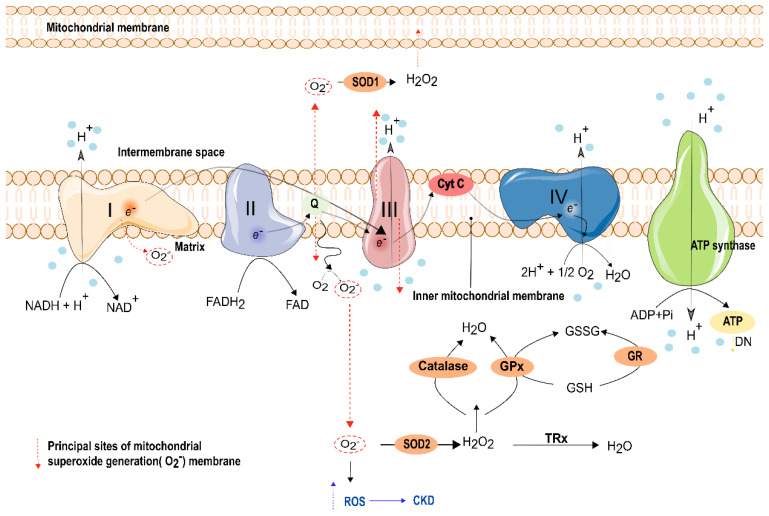
Mitochondrial oxidative phosphorylation and the mechanism of the main antioxidant enzymes present in the kidney. Reducing equivalents (NADH, FADH_2_) produced through the Krebs cycle donate electrons to the electron transport system at complex I and complex II. These electrons are subsequently transferred to other electron carriers, including coenzyme Q, complex III, cytochrome C (Cyt C), and complex IV. In complex IV, the oxygen is reduced into water. The donation of electrons provides the energy to pump protons (H^+^), which is responsible for the establishment of the electrochemical proton gradient at the inner mitochondrial membrane. This phenomenon generates the proton motive force that forces the protons back inside the matrix at ATP synthase to regenerate ATP from ADP and Pi. In diabetic nephropathy (DN), the production is reduced. With physiological conditions, some part of the O_2_ is converted into reactive oxygen species (ROS), where complexes I and III are considered the principal sites of ROS production. In the kidney, when the electron transport chain and mitochondria functioning is impaired, there is an imbalance between ROS and antioxidant activity, which may be a potentiator and contributor of kidney-related diseases such as chronic kidney disease (CKD), and DN. The main antioxidant enzymes are superoxide dismutase (SOD), catalase, glutathione peroxidase (GPx), and glutathione reductase (GR). SOD1 in the intermembrane space and SOD2 is released to the matrix. Levels of H_2_O_2_ are reduced to water through the cooperation between the antioxidant enzymes schematically represented in the scheme.

Electron transfer at complexes I and III has been suggested as the principal source of ROS overproduction [211] (Figure 3). In addition, several studies have been conducted to evaluate mitochondrial dysfunction as a potentiator and contributor to AKI [212]. Indeed, Emma and collaborators reported a decrease in ATP production related with an increase in ROS production, a release of Cytochrome C, and a disruption of mitochondrial cristae [213]. Histologically, a loss of cristae structure is also observed in AKI, leading to the dissipation of the mitochondrial membrane potential that consequently halted ATP production [213]. In line with these observations, several studies in animal models demonstrated the loss of mitochondrial respiratory proteins, particularly Peroxisome proliferator-activated receptor-gamma coactivator-1 α (PGC1-α) [214]. As expected, renal mitochondrial and ATP production are altered by diabetes. Curiously, early in diabetes, ATP generation is increased, yet it decreases as the disease progresses [215,216,217]. Oyarzún and collaborators identified an alteration of adenosine signaling in renal cell dysfunction. Here, in early diabetic rats, the authors observed an increased expression of the ecto 5′-nucleotidase (CD73), which hydrolyzes AMP to adenosine, at the renal proximal tubules, leading to tubulointerstitial fibrosis initiation [218]. Thus, higher levels of CD73 lead to the production of high levels of adenosine and emerges as a new tool for the early diagnosis of tubular injury in DN, being less invasive than normal biopsies [218].

A dysfunctional Electron Transport Chain (ETC) undergoes an increase in glycolysis, where there is an increase of lactate and pyruvate levels, as well as hexokinase activity [219,220]. This suggests that the kidney, after injury, can maintain its function by altering its metabolic preferences [221]. Glucose oxidation is recognized as the golden pathway adopted for ATP production in the kidney. Within the kidney, glomeruli, podocytes [222], endothelial cells [223], and mesangial cells [216] are highly dependent on glucose oxidation for energy production yet can shuttle substrates such as lactate into oxidative energy production following the conversion to pyruvate. Nevertheless, the delivery of metabolic substrates for ATP production, such as fatty acids and ketones, are changed by diabetes [224]. Alterations in metabolic fuel sources in diabetes provide a great contribution to renal hypoxia. In diabetes, there are some animal studies that describe that renal sites are damaged by hypoxia. One example is a study done in 2017 in pre-diabetic pigs, in which was observed hypoxia and mitochondrial dysfunction in the medullary ascending loop of Henle [225]. Moreover, chronic hypoxia of the proximal tubules has been characterized as a final common pathway in the progression of CKD to ESRD [226]. 

Several studies already demonstrated that renal disease can have an origin in deficiencies in OXPHOS as a result of mutations in Coenzyme Q10 biosynthesis monooxygenase 6 [227], cytochrome C oxidase 10 [228], Pdss2 [229], and p-53-controlled ribonucleotide reductase [230], in human and animal models. To have a deeper knowledge of the degree that ETC is affected, the evaluation of defects in the activity of the mitochondrial complexes, individually, may contribute to understanding how mitochondrial dysfunction and renal impairment are possibly correlated.

Gene mutations that result in complex I deficiency are the most common oxidative phosphorylation disorders in humans [231]. Forbes and collaborators studied mice with a knockdown of the complex I gene, Ndufs6. Heterozygous mice, with an approximately 40% impairment of complex I activity in the kidney, had a kidney disease that precedes cardiac abnormalities, whereas homozygous mice died of cardiomyopathy and advanced chronic kidney disease [232]. In line with these studies, complex I dysfunction was already identified in humans with diabetes. Here, it was observed a reduction by approximately 50% in complex I renal cortical activity [233]. Complex III deficiencies, associated with increased ROS production, were also detected in the diabetic kidney [234]. It is not a new theme of debate that mitochondria network changes, including bioenergetic alterations, are implicated in DN. Coughlan and collaborators, in a study of diabetic rat mitochondria, at four weeks of age, reported that these rats revealed a decrease in ATP content and mitochondrial fragmentation in the kidney, within proximal tubule cells. By eight weeks of age, they detected a generation of glomerular damage, due to an increased capacity for mitochondrial capacity. Later, worsened effects were observed. At 16 weeks of age, there was tubular damage and mitochondrial uncoupling. This study unveils that changes in mitochondrial bioenergetics and dynamics may precede the development of renal lesion in diabetes and not the other way around [235]. To complement this idea, a study in nondiabetic mice demonstrated that increasing kidney metabolism is not sufficient to induce kidney damage as it is in the presence of hyperglycaemia and hypertension [236].

Furthermore, complex I deficiency is also associated with an increased mitochondrial superoxide production in skin fibroblast mitochondria. Skin fibroblasts, obtained from young individuals with DN, also have mitochondrial dysfunction [237]. In mitochondrial ATP generation, the electron can leak from the respiratory chain and form superoxide, through the binding with oxygen [198]. Indeed, Complex I and Complex III are the main sites of superoxide production in renal mitochondria. Complex I generated superoxide is released to the mitochondrial matrix, while complex III generated superoxide is released to the matrix and intermembrane space [238,239] (Figure 2). Coenzyme Q10 (CoQ10) plays a crucial role in the mitochondria respiratory chain and protects against damage from ROS [240,241,242]. CoQ10 deficiencies have been correlated with severe renal pathologies, including glomerular and tubular interstitial disease [227,243,244]. Mutation in genes related to the biosynthesis of CoQ10, such as PDSS2, COQ2, COQ6, COQ8, and COQ9, have been related to the genetic nephrotic syndrome in humans [227,242,243]. Regarding COQ2 nephropathy, a study done in the Drosophila model revealed that the silencing of genes related to Q10 resulted in multiple abnormalities. Coq2-deficient nephrocytes showed elevated levels of autophagy and mitophagy that increased the levels of ROS. It also decreased respiratory chain activity at complexes II and III [242]. Additionally, disturbed regulatory miRNAs, such as miR21, were reported in a patient that carried an impaired IV activity and with CKD [245,246]. These studies highlight that mitochondria are intimately related to renal diseases. 

Urinary markers of mitochondrial dysfunction were evaluated in an individual with DN. This study quantified 94 urine metabolites in patients with diabetes mellitus (DM), and patients with CKD and DM, and in healthy controls. Thirteen metabolites were significantly reduced in DM+CKD patients, in which 12 of these 13 are linked to mitochondrial metabolism demonstrating a global suppression of mitochondrial activity in DN. Additionally, urine exosomes from patients with diabetes and CKD had a lower expression of PGC1-α (a master regulator of mitochondrial biogenesis) and presented less mitochondrial DNA [226]. PGC1-α is considered a booster for mitochondrial biogenesis [247] and is a key player in fuel switching [248]. An experimental model of kidney-related disease [214] demonstrated that the increase of PGC1-α activity is associated with renoprotection and mitochondrial biogenesis. Proximal tubules exposed to a diabetic environment, overexpression of the GTPase repressor/activator protein 1 homolog (RAP1) improved mitochondrial function through PGC1-α activation [249]. Additionally, overexpression of PGC1-α protected renal tubular cells against oxidant stress, with an increase in mitochondrial number and respiratory capacity [250].

As expected, Mitophagy is also altered in ischemic AKI. Mitochondria are dynamic organelles that constantly undergo fission and fusion, in response to cellular energy requirements and to maintain mitochondrial integrity [251]. Furthermore, the fusion of mitochondria is also a mechanism to overcome damage, by acquiring healthy proteins to improve their function [252]. During fission, mitochondria are split into two, where fusion is the combination of two mitochondria, to produce one larger organelle [251]. Succinctly, the fission process is mediated by dynamin-related protein (Drp1) and its receptors which allow for it to be properly recruited to the outer mitochondrial membrane to carry out fission, such as mitochondrial fission 1 (FISP1). Moreover, fusion is mostly regulated by the interaction between mitofusin 1 and 2 (MNF1/2). MFN1 and MFN2 are located on the outer mitochondrial membrane and are a key player for outer membrane fusion. Protein dynamin (also known as OPA1) resides in the inner membrane and is important for inner membrane fusion [253]. Dysregulation on these processes results in an impairment of ATP production [254]. Moreover, the NAD^+^-dependent SIRT family deacetylases, particularly the mitochondrial matrix-resided SIRT3 protein, play an important role in regulating mitochondrial dynamics and function under AKI [255]. Yet, the underlying mechanism of mitochondrial dysfunction in renal fibrosis warrants further research.

## 9. Antioxidant Capacity in the Kidney

Oxidative damage in the mitochondria alters DNA transcription, which affects the normal functioning of mitochondrial proteins, antioxidants, and oxidative phosphorylation enzymes [256]. This OS-induced injury may be derived from ischemia/reperfusion, energy shortage from impaired biogenesis, ATP energetics, or even defects in the mitophagy process [190]. 

The imbalance between the generation of ROS levels and local antioxidant capacity is a biomarker of mitochondrial dysfunction in DN [217,235]. To overcome the excessive production of ROS, mitochondria have an antioxidant system. Indeed, homeostasis is maintained through a complex set of antioxidantal mechanisms that prevent oxidative stress-induced injury (Figure 3). The physiologic formation of ROS is detoxified by endogenous antioxidants, which are classified into two groups: enzymatic and non-enzymatic. Enzymatic antioxidants include superoxide dismutase (SOD), catalase, glutathione peroxidase (GPx), glutathione S-transferase (GST), and glutathione reductase (GR), while non-enzymatic pathways include ETC, glucose autoxidation, glycation products (AGEs), GSH, and other pathways [257]. Additionally, exogenous antioxidants obtained from daily food intake can be hydrophilic (Ascorbate/vitamin C and flavonoids) or lipophilic (α-tocopherol/vitamin E and carotenoids), being classified as nonenzymatic antioxidants [258]. Under physiological conditions, mitochondria have an efficient antioxidant system to cope with ROS [259], which includes, superoxide dismutase that reduces O_2_^−^ in H_2_O_2_ faster than the rate of O_2_^−^ production [168]. Levels of H_2_O_2_ lead to glutathione peroxidase activation which reduces H_2_O_2_ into water (Figure 3) [260]. Increased levels of ROS have multiple consequences. It can cause breaks in mitochondrial DNA, which leads to mutations in the next generation, and it can also trigger apoptosis (such as Cytochrome C) [261]. In an OS situation, NRF2 can boost an answer to ROS overproduction [261]. The association of Nrf2 with kidney disease has been reported in some studies. In mice with DN, Nrf2−/− showed increased ROS levels and higher oxidative DNA damage. Along to this, increased TGF-β1 and ECM accumulation were found. These aspects were all decreased when Nrf2 was activated, which significantly prevented the progression of AKI to CKD transition [262].

Nrf2 can activate transcription of heme oxygenase 1 (HO-1), and ferritin that can mitigate AKI and renal injury, on glomerular endothelium, cortical peritubular capillaries, and interstitial leukocytes [263]. Natural bioactive compounds demonstrated kidney protective effects by activating Nrf2 in experimental CKD models [264]. HO-1 inducers were applied in multiple diseases, including CKD. Sulforaphene, an Nfr2 activator, was able to reduce ROS induced by PI3K/Akt/GSK3β activity and EMT, in HK-2 cells, after high glucose exposure [265]. Again, in a high glucose situation, Astaxanthin attenuated OS and fibronectin accumulation in glomerular mesangial cells and improved the metabolic performance and kidney morphology in STZ-induced diabetic rats [266].

Furthermore, curcumin was able, through activation of Nrf2 signaling, to ameliorate albuminuria levels, in a rat model of CKD, by attenuating inflammation [267]. In another study, curcumin was also suggested to prevent EMT of podocytes, proteinuria and kidney injury in DN [268]. In line with this study, 14 randomized controlled studies demonstrated that curcumin markedly lowers mesangial area, proteinuria, serum creatine, and protects the kidney of rats or mice with diabetes [269].

Silibin was also able to induce biochemical changes in the kidney of rats, by reducing lipid peroxidation and improved antioxidant defense that contributed to the preservation of the normal histological architecture of the renal tissue [270].

Factors that cause OS are activation of NADPH oxidases, uncoupled endothelial nitric oxide synthase, and mitochondrial dysfunction together with decreases antioxidant defenses, such as a decreased expression of antioxidant enzymes and intracellular GSH content. 

The glutathione redox cycle is the critical antioxidant mechanism found in several intracellular organelles, such as mitochondria [260]. Mitochondrial glutathione (mGSH) can be oxidized to glutathione disulfide (GSSG) by superoxide anions [260] (Figure 3). In addition, GSSG can be reversed again in mGSH, through glutathione reductase, which requires NADPH from the pentose phosphate pathway. To maintain the oxidative balance in the cells, the cell must contain low levels of GSSG and high levels of GSH [201]. Furthermore, severe OS can overcome the ability of the cell to reduce GSSG to GSH, resulting in the accumulation of GSSG [271]. Hence, the ratio of GSH and GSSG is a marker of OS [272]. Furthermore, mutual maintenance of ascorbate and GSH can occur in vivo, since ascorbate can maintain intracellular GSH [273]. Thus, these mechanisms are considered pivotal process for preserving mitochondrial functions.

An increasing number of studies demonstrated that enhancement of antioxidant enzyme activity resulted in a protective effect in animal models of AKI [274]. SOD is a key enzyme in the detoxification of free radicals and all three isoforms are highly expressed in the kidney [275]. It converts O_2_^−^ to H_2_O_2_ and oxygen, and in turn, the catalase or glutathione peroxidase system reduces H_2_O_2_ to water [201]. Administration of SOD or catalase can diminish ROS in proximal tubule injury after hypoxia in vitro [276]. It can also attenuate oxygen radicals in vivo after renal ischemia in rabbits [277]. In rats, SOD was able to improve renal function and reduced tissue injury and cortical mitochondrial lipids peroxidation [278]. Long-term treatment with a SOD mimetic (MnTMPyP) alleviated oxidation parameters in kidney fibrosis ischemic [279]. MnTMPyP blocked O_2_^−^ and peroxynitrite formation, and reversed functional kidney deficits, suggesting that antioxidant intervention is beneficial, and halting ROS formation can alleviate microvascular failure and renal injury [280]. Another similar study is in early experimental DM in hypertensive rats, where the administration of tempol, another SOD mimetic, corrected the oxidative imbalance and improved oxidative stress-induced renal injury, decreasing albuminuria and fibrosis [281].

Decreases in renal superoxidase are accompanied by mitochondrial biogenesis and less mitochondrial glucose oxidation in diabetic mice [282]. Mitochondrial biogenesis can occur as a cellular response to oxidative stress [283]. Regarding the progression of DN, the contribution of mitochondrial superoxide production is not clear. However, some data led to the theory that superoxide production can be an indicator of mitochondrial healthy activity [284].

There is no doubt that hyperglycemia causes high production of ROS. Hence, future studies are needed to provide a better insight into the diabetic kidney. Indeed, particularly in this class of patients, where albumin is no longer considered a biomarker of the disease, there is an urgent need to find, if possible, a new biomarker, especially at earlier stages, to inhibit the onset and the progression of DN. In recent years, mitochondria-target therapeutics have attracted much interest as they demonstrated that attenuating mitochondrial dysfunction can prevent the progression of kidney diseases [190,285].

## 10. Targeting Kidney Mitochondria—Therapeutic Approaches

Mitochondria-targeted antioxidants are based on the mechanism of delivery of known redox agents to the mitochondrial matrix through conjugation with TPP+ (triphenylalkyphosphonium cation) moiety [286]. As aforementioned, CoQ10 has renoprotective and prevents detrimental changes in mitochondrial function and morphology. A derivative of CoQ10, known as mitoquinone (MitoQ), is studied in multiple clinical trials, including Parkinson′s disease [287] and CKD [288]. This molecule could be delivered and concentrated at mitochondria matrix to function as ROS scavenger [286]. Administration of MitoQ mitigated mitochondrial OS that resulted in the prevention of DN in a murine model of inherited diabetes [288] and experimental models T2DM [289]. These studies revealed an improvement of renal function, a decreased glomerular hyperfiltration, and albuminuria. MTP-131, a cationic peptide with antioxidant properties that increases the efficiency of OXPHOS, demonstrated an improvement in kidney function and fibrosis in models of AKI [289], and diabetes [290]. Recently, its efficiency on mitochondrial myopathies is being tested in Phase I and II clinical trials [188,291]. 

Furthermore, MitoTEMPO is another selective mitochondria-targeted antioxidant that could be uptaken and accumulated in the energized mitochondria matrix and hence, modulate CoQ10. Sun and collaborators developed a study where they co-administrated MitoTEMPO (3 mg/kg/day) with CoQ and ML385 (an antagonist, used to inhibit Nrf2) (30 mg/kg/day) in db/db mice. Here, they observed a restoration of the mitophagy mechanism (through the activation of the Nfr2/ARE pathway) and alleviation of kidney dysfunction in glomeruli [292]. In line with these studies, in another study performed in nephrectomy mice, MitoTEMPO administration (1 mg/kg/day), was able to improve renal function and alleviate renal fibrosis by reducing cytokines, mitochondrial dysfunction, ER stress, and profibrotic factors through the Sirt3-SOD2 pathway [293]. Surprisingly, renal MnSOD inactivation associated with increased mitochondrial O_2_^−^, can be attenuated with the mitochondrial-targeted antioxidant Mito-TEMPO [294].

Mito-CP is a SOD mimetic that targets mitochondria and protects against cell dysfunction, apoptosis, injury, and inflammation in mice administered cisplatin. Additionally, it reduced NOX2/4 mRNA and protein, lipid oxidation, protein nitration, and pro-inflammation markers (MPO, ICAM-1) [295]. Moreover, mitochondrial-targeted peptides protected cardiolipin from cytochrome C peroxidation, demonstrating efficacy against oxidative stress, tubular cell damage, and dysfunction in IRI [296].

Edaravone (MCI-186) is a small molecular weight compound that in rats was able to attenuate ROS radical generation in kidney tubular cells in vitro, lipid peroxidation, and mitigated renal dysfunction in IRI [297]. In dogs, similar results were observed, in which there were improvements in renal function and reduced renal tubular cell damage, lipid, and oxidation [298]. 

NAC is a synthetic derivative of cysteine and precursor of GSH that revealed to be protective in the Rhabdomyolysis (RM)-induced AKI animal model [299] improving kidney function, renal GSH, and systemic oxidative stress. However, in some clinical trials in humans, no positive effects were achieved in AKI, due to its heterogeneity and to the confounding serum creatinine levels as possible contributors to the neutral effects [300]. 

Another possible approach is to benefit from dietary antioxidants. For example, vitamin E and selenium attenuate nephrotoxicity [301]. Surprisingly, selenium, which can enhance the activity of GSH-dependent antioxidant enzymes, and hence can inhibit renal oxidative damage, had no renoprotective effects in an animal model of RM-mediated AKI [302]. Vitamin C, also known as ascorbate, is also able to attenuate oxidative damage and renal injury in some animal models [300]. It has multiple actions in human renal injury, such as renoprotection in AKI, a reduction in multi-organ failure, an improvement in renal function in sepsis and severe burns and enhancement of endothelial-dependent vasodilation in renal allograft [274]. 

Vitamin C is an essential nutrient obtained from the diet and is a highly effective non-protein reducing agent capable of donating electrons in widely enzymatic and non-enzymatic reactions [303]. Interestingly, vitamin C can be recycled via GSH and /or GSH or NADH-dependent enzymes, enhancing the potential protective action of this vitamin [273]. Thus, vitamin C is proposed to have an important physiological role as an effective in vivo antioxidant once it has a low reduction potential, which allows direct interaction with a wide range of physiological ROS/RNS [304]. Vitamin C scavenges radicals (O_2_^−^) and non-radical oxidants and reduces levels of α-tocopheroxyl radical in lipids and membranes, allowing recycling of vitamin E and inhibition of lipid peroxidation [305]. Another positive effect is that it can be administrated in large quantities, with minimal adverse effects [306,307]. Low plasma vitamin C is a risk for mortality in patients with AKI co-morbidities, such as diabetes [308]. Groebler and collaborators compared treatment with vitamin C alone or in combination with a synthetic polyphenol. Both compounds decreased plasma and kidney oxidative markers when administrated alone or in combination. Nevertheless, only vitamin C revealed a potential clinical effect on reducing proteinuria, plasma urates, and renal tubule casts [309].

These results demonstrate that vitamin C intake may prevent ROS-mediated renal damage in AKI. 

## 11. Conclusions

Interstitial fibrosis is the most common outcome of all forms of CKD. Given the high morbidity and mortality associated with AKI and CKD, there is an important unmet medical need to recover renal function. There is no doubt that it is necessary to pay better attention to the condition of diabetes, even more when this pathology is associated with kidney problems, as these patients end up having a clinical worsening much sooner. Here, diabetic nephropathic patients deserve better attention, as their diagnosis fails in the sense of being early, due to the lack of biomarkers. Henceforth, it is important to find new disease markers, preferably at earlier stages. Mitochondria seems to be a plausible way forward, as deficiencies at various levels have been shown to be indicators of the development of kidney disease. The correction and or the prevention of mitochondrial dysfunction may be pivotal in the prevention and treatment of kidney disease. Therefore, it may be prudent to consider ROS levels as an indicator of mitochondrial function rather than an endpoint marker of damage. Cells have their own antioxidant defense system against multiple disease, where redox imbalance has a critical role in the pathogenesis of CKD progression. This disturbance already starts in an early phase of CKD, and so, intervention at this point can help to attenuate deleterious effects. Many promising approaches are currently being investigated. Thus, it is necessary to develop a deeper knowledge, to detect whether it is a structural abnormality that trigs the gradual loss of renal function, or if it is a bioenergetic issue. Hence, the detection of mitochondrial dysfunction, both in structure or in terms of complexes activity, may promote the development of a more personalized therapy, which can act at the level of a single complex, or in a specific protein to improve renal function. Likewise, it is needed to investigate the detailed molecular mechanism of the renoprotection of the kidney, such as new advances in drug delivery systems, in order to avoid the poor bioavailability of some pharmacologic approaches. It should be considered to give the mitochondria a more important role in kidney disease prognosis since it seems to be an attractive approach given to their possibility of targeted therapy, than the currently used.

## Figures and Tables

**Table 1:** Therapeutic options available to delay kidney fibrosis.

Therapeutic Options		Target	Function	Effect	References
LY2382770 (phase II study)		TGFβ-1	Modulator of TGFβ-1	-	[35]
Barcitinib		JAK-STAT pathway	↓ JAK-STAT pathway	↓proteinuria levels	[35]
FG-3019		CTGF	Modulator of TGFβ-1	↓microalbuminuria	[91]
Triterpenoid compounds (PZC, PZD, PZE)		RAS	Smad3 signaling and TGFβ-1 signaling	↓ TGFβ-1	[95]
ACE2		TGFβ-1 with Smad3 signaling	Degrade Angiotensin II	↓renal fibrosis ↓albuminuria ↓glomerular hypertrophy	[98]
Imatinib		RTK	PDFGR inhibitor	↓proliferation of fibroblasts	[110]
AG1296		RTK	FGFR blockage	-	[111]
Losartan		RAS	Angiotensin II	Delay of DN	[112]
Captopril					[113]
Liraglitude semaglutide		GLP-1	insulin secretion and suppression of glucagon	↓albuminuria ↑GFR	[117,118,119]
Empagliflozin		SGLT2	SGLT2 inhibition	Delay the advance of renal disease	[123]
PBI-4050		FFA1R	Anagonist of FFA1R	↓renal fibrosis	[125]
C75		CPT1	CPT1 activator	↓renal fibrosis	[126]
Atrasentan		ET_A_ blockers	-	↓renal fibrosis ↓albuminuria	[131]
Avosentan					[132]
Emodin		-	-	↓ fibronectin ↓ TGFβ-1	[135]
Celastrol		Smad3	↑cannabinoid receptor 2 expression	↓renal fibrosis	[138]
microRNAs	siRNA	Smad4	Silecing of Smad4	↓fibrosis ↓ α-SMA expression	[147]
	miR-21 miR-29 miR-192	TGF-β1/SMAD signaling	Smad3 inhibitors	↓collagen deposition ↓ fibronectin ↓α-SMA expression	[148,149,150]
	miRlet7c	-	-	↓fibrosis ↓ TGFβ-1 ↓MMP-9	[153,154]
hUCB-SC		-	-	↓ fibronectin ↓α-SMA expression ↓proteinuria levels ↓E-cadherin	[157]

Abbreviations: TGF-β1—transforming growth factor beta; α-SMA—alpha smooth muscle actin; MMP-9—matrix metalloproteinase; DN—diabetic nephropathy; RAS—renin-angiotensin system; ACE-ACE2—angiotensin-converting enzyme; CTGF—Connective Tissue Growth Factor; JAK-STAT—Janus kinase-signal transducer and activator of transcription pathway; RTK—Receptor of tyrosine kinase; PDFGR—platelet-derivated growth factor receptor; FGFR—fibroblast growth factor receptor; SLGT2—Sodium glucose transporter 2; FFA1R—Free fatty acid 1-receptor; CPT1—carnitine *O*-palmitoyltransferase 1; ETA—Endothelin receptor; GLP-1—Glucagon-like receptor-1; GFR—Glomerular Filtration Rate; ↑—increased; ↓—decreased; - data unavailable.

## Data Availability

Not applicable.
